# GPR83 protects cochlear hair cells against ibrutinib-induced hearing loss through AKT signaling pathways

**DOI:** 10.3389/fmed.2025.1579285

**Published:** 2025-04-03

**Authors:** Yuhua Zhang, Yun Xiao, Yongjun Zhu, Lin Yan, Nan Cheng, Yongjie Wei, Yanling Zhang, Yanghua Tian, Wei Cao, Jianming Yang

**Affiliations:** ^1^Department of Otolaryngology-Head and Neck Surgery, The Second Affiliated Hospital of Anhui Medical University, Hefei, China; ^2^School of Life Sciences, Anhui Medical University, Hefei, China; ^3^Department of Neurology, The Second Affiliated Hospital of Anhui Medical University, Hefei, China

**Keywords:** ibrutinib, hearing loss, hair cell, G protein-coupled receptor, Z-VAD-FMK, apoptosis

## Abstract

**Introduction:**

Ibrutinib, widely used in leukemia treatment, has been implicated in sensorineural hearing loss; however, its underlying mechanisms remain unclear.

**Methods:**

This study investigated the impact of ibrutinib on hearing using HEI-OC1 cells, cochlear explants and C57BL/6 J mice. We used RNA-sequences analysis to investigate the potential mechanisms of ibrutinib-induced ototoxicity. Mice received ibrutinib and auditory thresholds were assessed via auditory brainstem response testing; to assess the potential protective effects, we co-administered the caspase inhibitor Z-Val-Ala-Asp (OMe)-fluoromethylketone (Z-VAD-FMK) and monitored hearing.

**Results:**

Z-VAD-FMK mitigated ibrutinib-induced hearing loss by inhibiting apoptosis in auditory cells. Ibrutinib exposure resulted in cochlear hair cell (HC) damage and subsequent hearing loss by inhibiting the protein kinase B and G protein-coupled receptor 83 (GPR83) pathways. RNA sequencing suggested that GPR83 protects HCs by modulating autophagy. Z-VAD-FMK application and GPR83 overexpression attenuated ibrutinib-induced cochlear HC apoptosis and auditory decline.

**Conclusion:**

These findings confirm ibrutinib’s ototoxicity and highlight the protective role of GPR83 in ibrutinib-induced hearing loss, supporting future clinical investigations into Z-VAD-FMK and GPR83 as interventions for ibrutinib or other chemotherapeutic drug-induced ototoxicity.

## 1 Introduction

Deafness affects approximately 466 million individuals globally (1). Genetic predisposition, aging, and exposure to loud noises increase the risk of hearing loss. As hearing loss severely impacts communication, social participation, and overall quality of life, developing effective preventive strategies to mitigate the incidence of drug-induced hearing impairment is essential.

Certain chemotherapeutic medications can induce hearing loss by harming the cochlear hair cells (HCs) (2). Ibrutinib is an orally administered tyrosine kinase inhibitor commonly used to treat B-cell lymphoma and chronic lymphocytic leukemia (3). Ibrutinib selectively inhibits phosphoinositide 3-kinase (PI3K) in malignant cells while sparing normal cells (4, 5). Additionally, it inhibits epidermal growth factor receptor (EGFR) activity and induces apoptosis in tumor cells (6). Although ibrutinib is generally associated with low toxicity, clinical trials have reported a significant association between its use and an increased risk of hearing impairment (7, 8). However, the underlying molecular mechanisms of ibrutinib in causing hearing loss remain unclear.

G protein-coupled receptors (GPCRs) are the primary cellular signaling sensors in human cells, comprising over 800 members. These receptors play a pivotal role in regulating various physiological processes. For example, these receptors detect extracellular signals and activate intracellular G proteins, mediating signal transmission that regulates cellular functions and phenotypes (9, 10). GPR83, an orphan GPCR protein family A receptor, is widely expressed in the brain regions associated with energy metabolism (11). GPR83 potentially plays a role in nociceptor function, which may be associated with pain perception (12). Some GPCRs may be involved in several disease-causing mechanisms related to cochlear hearing loss, including genetic factors, noise, ototoxic drugs, and cochlear structural changes (13–17). Moreover, certain GPCRs inhibit hearing impairment by decreasing reactive oxygen species (ROS) in the cochlea (18).

Chemotherapeutic drugs can increase ROS levels in the cochlea, which can elevate AKT levels and activate caspase-3, leading to a cascade of cellular events culminating in programmed cell death (19–21). Several studies have implicated the caspase-dependent pathway as a key mediator of drug-induced cochlear HC loss (22–25). Cysteine-aspartic acid proteases belong to the caspase-3 family, regulate apoptosis in multicellular organisms, and play a pivotal role in cochlear cell apoptosis and HC damage (26). However, the activation of caspase proteases can be inhibited by the synthetic peptide Z-Val-Ala-Asp(OMe)-fluoromethylketone (Z-VAD-FMK), which binds irreversibly to the active site of caspase-3 (27). Blocking the caspase pathway can protect cochlear cells from apoptosis induced by various factors (28, 29). Consequently, Z-VAD-FMK has been increasingly used to treat cochlear damage caused by increased caspase activity owing to noise exposure or aging (30–32). This raises the hypothesis that Z-VAD-FMK might also mitigate hearing impairments caused by ibrutinib therapy.

This study aimed to investigate the ototoxic mechanisms of ibrutinib, focusing on the protective role of Z-VAD-FMK against HC damage, synaptic loss, and decline in auditory function.

## 2 Materials and methods

### 2.1 Animal drug administration experiments

Six-week-old C57BL/6 J mice were maintained at the Core for Laboratory Animal Medicine, Institute of Health and Medicine, Hefei Comprehensive National Science Center, under standard conditions (12 h light/dark cycle, temperature 22 ± 2°C, and humidity 40–60%). All animal procedures were conducted in accordance with the guidelines of the Institute’s Animal Care and Use Committee (IACUC) and approved by the Hefei Comprehensive National Science Center Institutional Animal Ethics Committee (approval number: IHM-AP-2024-032). Twenty mice were randomly allocated into four groups. Each group was subjected to a 15-day treatment schedule. Group 1 (the control group) received daily injections of an equivalent volume of saline. Group 2 was administered 50 mg/kg ibrutinib orally once daily, as previously described (33). Group 3 received daily intraperitoneal injections of 50 mg/kg ibrutinib only, whereas Group 4 mice received daily treatment with ibrutinib, along with subcutaneous injections of 100 mg/kg furosemide. In the Z-VAD-FMK prevention study, the mice were administered 2 mg/kg Z-VAD-FMK (S7023, Selleck, Houston, TX, United States) as a pre-treatment 2 h before ibrutinib administration.

### 2.2 Cell culture and viability assay

HEI-OC1 cells (HC-like cell line) were cultured in Dulbecco’s Modified Eagle Medium (DMEM; C11995500BT, Gibco, Waltham, MA, United States) with 10% fetal bovine serum (03.U16001DC; EallBio, Ashford, United Kingdom) and 50 μg/mL ampicillin (A105484; Aladdin). When the cultured cell vessel reached 90% confluency, 2 × 10^3^ cells per well were implanted into 96-well plates after being detached using 0.25% trypsin-ethylenediaminetetraacetic acid. Z-VAD-FMK (5, 10, 20, and 50 μM) or ibrutinib (0.5, 1, 5, and 10 μM) were applied to the HEI-OC1 cells for 24 h after overnight incubation. Subsequently, 10 μL of cell counting kit-8 (CCK-8) solution (HY-K0301; MedChemExpress, Monmouth Junction, NJ, United States) was introduced to the cell cultures, which were maintained at 37°C for 30 min. The absorbance of the CCK-8 cells was measured using a microplate reader (BioTek Synergy HTX, Agilent, United States).

### 2.3 Cochlear explants culture

Cochlear tissue samples were removed from the temporal bone of the C57BL/6 J mice on postnatal day 3 (P3) and cultivated on Cell-Tak-coated slides (354240; Corning, Corning, NY, United States) in a solution comprising DMEM (97%), B-27 supplement (2%) (17504044; Gibco), N-2 supplement (1%) (A1370701; Gibco), and ampicillin (50 μg/mL). The cochlear explants were subjected to a 12 h recovery incubation period, followed by treatment with either ibrutinib (0–70 μM) or Z-VAD-FMK (0–200 μM) for 24 or 16 h.

### 2.4 RNA extraction and RT-qPCR

TRIzol Reagent (15596–018; Life) was used to isolate RNA from both HEI-OC1 cells and the cochlea. cDNA synthesis reagent (K1622; Thermo Fisher Scientific) was used to reverse transcribe the extracted RNA. RT-qPCR was performed using a Bio-Rad Applied Biosystems CFX96 Real-Time PCR System (Bio-Rad, Hercules, CA, United States). The cycling conditions were as follows: initial denaturation at 95°C for 15 s, followed by 40 cycles of denaturation at 95°C for 15 s, annealing at 60°C for 15 s, and extension at 72°C for 20 s. The mRNA data were standardized relative to the GAPDH gene using the comparable cycle threshold technique (△△Ct). The primer sequences used are listed in the Supplementary Table S1.

### 2.5 Terminal deoxynucleotidyl transferase dUTP nick-end labeling assay

After 16 h of ibrutinib exposure, the HEI-OC1 cells and cochlear explants were fixed using 4% paraformaldehyde (P1110; Solarbio, Beijing, China). Apoptosis was measured using a dUTP nick-end labeling (TUNEL) BrightRed kit (A113; Nanjing, China, Vazyme). The specimens were treated with equilibration buffer (1×) at room temperature for 30 min and were then exposed to 50 μL of labeling buffer comprising double distilled water, recombinant TdT enzyme, 5× equilibration buffer, and BrightRed labeling mix and incubated for 60 min at 37°C. After three washes with phosphate-buffered saline (PBS), the nuclei were stained with 4′,6-diamidino-2-phenylindole. Myosin7a antibody (25–6790; Proteus Biosciences, Ramona, CA, United States) was used to stain HCs. Images of the HEI-OC1 cells and cochlear explants were captured using a confocal microscope (LSM880; Carl Zeiss, Oberkochen, Germany).

### 2.6 Western blotting

Proteins were obtained from cochlear samples or HEI-OC1 cells using radioimmunoprecipitation assay lysis buffer (P0013C; Beyotime, Shanghai, China) and protease inhibitors (4693132001; Roche, Basel, Switzerland). Total protein concentration in the samples were estimated using the bicinchoninic acid assay kit (P0010; Beyotime) according to the manufacturer’s guidelines. Subsequently, sodium dodecyl sulfate-polyacrylamide gel electrophoresis was performed using approximately 30–40 μg of protein extracted from each group. The separated protein bands were immunoblotted onto a polyvinylidene fluoride (PVDF) membrane and time-blocked using 5% nonfat powdered milk for 1 h at 25°C. After three washes to eliminate the primary antibodies, the PVDF membrane was incubated with secondary antibodies for 2 h after being left overnight at 4°C. An enhanced chemiluminescence kit (34075; Thermo Fisher Scientific) was used to detect the target protein, and band intensities were analyzed using ImageJ software. GAPDH and alpha-tubulin (60004-1-Ig, 11224-1-AP; Proteintech, Rosemont, IL, United States) were used as loading controls. The cleaved caspase-3 (CASP3) antibody (#9661; Cell Signaling Technology, Danvers, MA, United States) was used as a marker of apoptosis. The PVDF membrane was also incubated with GPR83 polyclonal antibody (PA5-114742; Thermo Fisher Scientific), phosphor-EGF receptor (Tyr1068, D7A5, p-EGFR, 3777; Cell Signaling Technology), AKT antibody (9,272; Cell Signaling Technology), and phosphor-AKT (p-AKT, Ser473, 4,060; Cell Signaling Technology) to detect the activity of the related pathways.

### 2.7 Immunocytochemistry staining

The HEI-OC1 cells and cochleae were immobilized in a 4% paraformaldehyde solution for 1 h at 20°C. They were then permeabilized for 15 min using 1× PBS (10010023; Gibco), treated with 1% Triton X-100 (T8787; Sigma-Aldrich, St. Louis, MO, United States), and blocked for 1 h with donkey serum (10%, SL050; Solarbio). The blocked cell samples were exposed to cleaved CASP-3 and myosin7a and incubated overnight at 4°C. Purified mouse anti-C-terminal binding protein 2 (CTBP2, 612004; BD Biosciences) was used to mark the ribbon synapses. Secondary antibodies including goat anti-rabbit Alexa Fluor Plus 488 (A32731; Thermo Fisher Scientific), goat anti-rabbit Alexa Fluor Plus 555 (A32732; Thermo Fisher Scientific), and goat anti-mouse IgG1 Alexa Fluor 568 (A21124; Invitrogen, Waltham, MA, United States), were used to amplify the signals. ROS and mitochondrial membrane potential in living cells was assessed using the Mitochondrial Superoxide (Mito-SOX) Indicator (M36008; Invitrogen) and tetramethylrhodamine ethyl ester (TMRE, C2001S; Beyotime). The cells were stained with TMRE or Mito-SOX, which were diluted in PBS for 10 min at 37°C according to the manufacturer’s instructions. Stained cells were viewed under a confocal fluorescence microscope (LSM880; Carl Zeiss, Oberkochen, Germany).

### 2.8 Auditory brainstem response measurement

The effects of ibrutinib administration on auditory function in mice were evaluated using auditory brainstem response (ABR) testing. The experiments were conducted in a specialized soundproof chamber. After administering 100 mg/kg pentobarbital for anesthesia, the mice were placed on a warmed pad set to 37°C. Electrodes were implanted in both the vertex and subdermal regions behind the ears of the mice. Neural responses to auditory stimuli at frequencies of 4, 8, 12, 16, 24, and 32 kHz were measured using electrodes and captured using a Tucker-Davis Technologies System III apparatus.

### 2.9 RNA sequencing

RNA was extracted to create libraries after treating cochlear explants with ibrutinib for 24 h. The stranded RNA sequencing libraries were extracted using 2 μg of total RNA with the KC Stranded mRNA Library Prep Kit (Illumina, San Diego, CA, United States) per the manufacturer’s instructions. PCR products spanning 200–500 bp were amplified and gauged before sequencing on a DNBSEQ-T7 sequencer using the PE150 model. The raw sequencing data were analyzed using Trimmomatic (version 0.36) to eliminate substandard readings and clip adapter sequences. The aligned data were then matched with the mouse reference genome using STRA software (2.5.3a) with default settings. Reads within the gene exon regions were quantified using FeatureCounts to obtain reads per kilobase of transcript per million mapped reads. Differential gene expression among the groups was evaluated using edgeR (version 3.12.1). A *p*-value threshold of 0.05 and a fold-change cutoff of 2 were used to identify significant differences between groups. Pathway enrichment analysis was conducted using KOBAS (version 2.1.1) to identify differentially expressed genes according to the Kyoto Encyclopedia of Genes and Genomes (KEGG). A significance threshold of 0.05 was used to discern the genes that were differentially expressed. Additional analysis was performed to identify alternative splicing occurrences using rMATS (version 3.2.5). A false discovery rate threshold of 0.05 and a minimum absolute delta psi (Δψ) value of 0.05 were applied.

### 2.10 Statistical analyses

Data were analyzed using GraphPad Prism 9. Continuous variables were characterized using means and corresponding standard deviations (SD). An analysis of variance was performed to investigate whether the groups had notable variations between them. Dunnett’s test was used to detect differences. Statistical significance was set at *p* < 0.05.

## 3 Results

### 3.1 Ibrutinib damages hair cell-like HEI-OC1 cells

**Figure 1:**
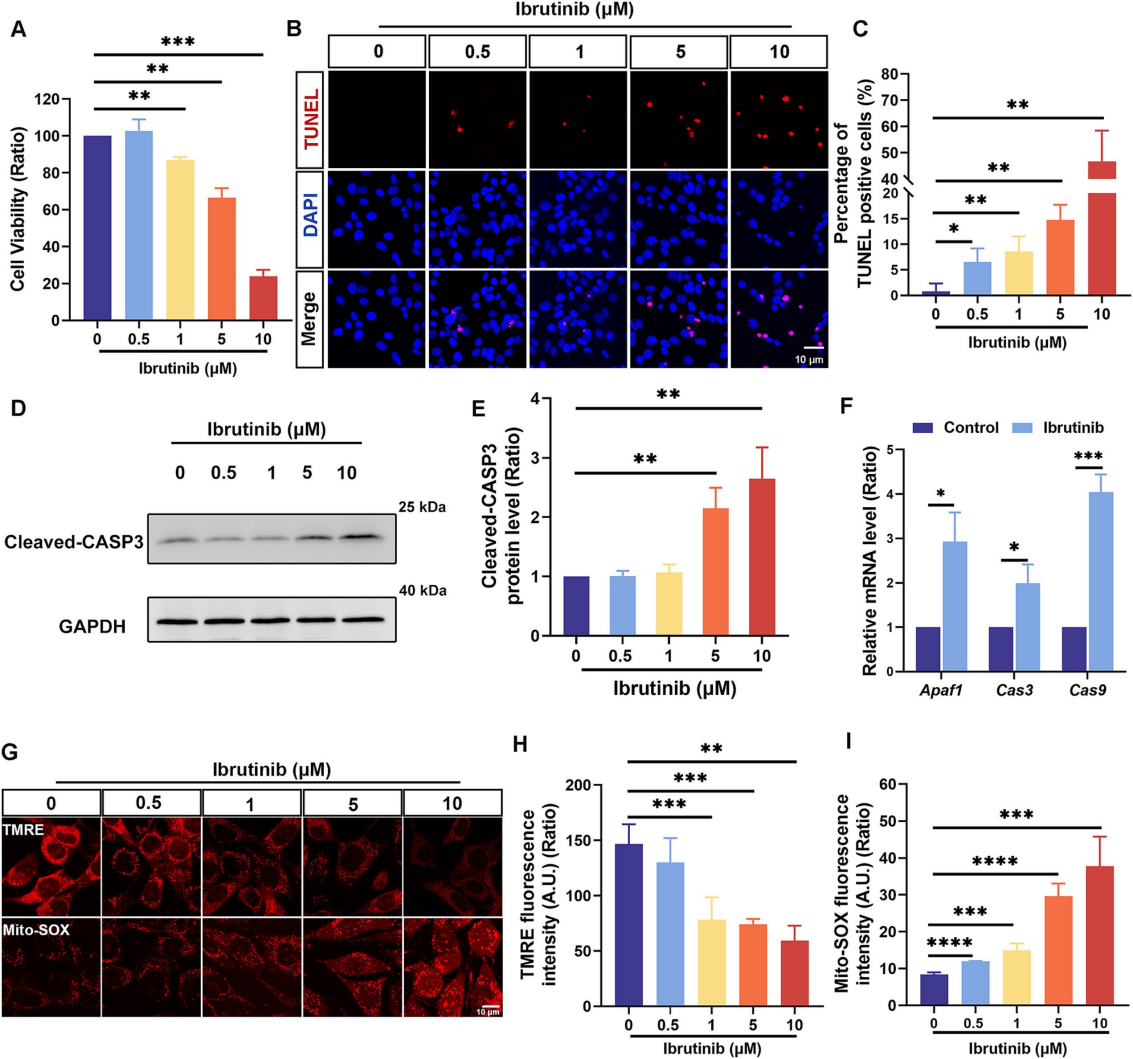
Apoptotic HEI-OC1 cells increased after ibrutinib treatment. **(A)** Assessment of cell survival through the cell counting kit-8 assay after exposing HEI-OC1 cells to several ibrutinib concentrations for 24 h, *n* = 3. **(B)** Detection of HEI-OC1 cell apoptosis after exposure to different ibrutinib concentrations for 16 h using DAPI and TUNEL staining. Scale bar: 10 μm. **(C)** The number of cells positive for DAPI and TUNEL staining from panel **(B)**, *n* = 4. **(D)** Analysis of cleaved caspase-3 (CASP3) expression changes in HEI-OC1 cells using western blotting. The cultured cells were exposed to different ibrutinib concentrations for 16 h. **(E)** Quantitative evaluation of the western blotting data from panel **(D)**, *n* = 5. **(F)** Examination of three apoptosis-related mRNA levels after treatment with 5 μM ibrutinib for 16 h through qRT-PCR, *n* = 3. **(G)** Alterations in mitochondrial membrane potential and oxidative stress after 24 h of ibrutinib treatment as indicated by tetramethylrhodamine ethyl ester (TMRE) and Mitochondrial Superoxide (Mito-SOX) staining. Scale bar: 10 μm. **(H)** Assessment of TMRE fluorescence intensity through statistical analysis, *n* = 3. **(I)** Statistical analysis of the Mito-SOX fluorescence intensity, *n* = 3. Data are shown as the mean ± standard deviation (SD). The significance degrees are denoted as follows: * *p* < 0.05, ** *p* < 0.01, *** *p* < 0.001, and **** *p* < 0.0001.

HEI-OC1 cells were analyzed to determine the effects of different ibrutinib concentrations. Ibrutinib had a substantial cytotoxic effect on cells depending on the dosage, particularly at concentrations >0.5 μM (Figure 1A). The toxicity of ibrutinib was confirmed by measuring the number of apoptotic cells using TUNEL staining (Figure 1B). Quantitative analysis demonstrated a positive correlation between the ibrutinib concentration and the apoptosis level in HEI-OC1 cells (Figure 1C). Western blot analysis revealed that the levels of CASP3 increased with increasing ibrutinib concentrations (Figures 1D,E). mRNA levels of genes associated with apoptosis were elevated in HEI-OC1 cells after exposure to 5 μM of ibrutinib for 16 h (Figure 1F). TMRE staining showed that cells treated with ibrutinib had significantly reduced mitochondrial membrane potential (Figures 1G,H). Moreover, Mito-SOX staining confirmed that HEI-OC1 cells treated with different ibrutinib doses exhibited high levels of oxidative stress (Figures 1G,I). These results support the hypothesis that ibrutinib damages HEI-OC1 cells, even at concentrations as low as 0.5 μM. Because only 60% of HEI-OC1 cells survived after treatment with 5 μM of ibrutinib, this concentration was selected for subsequent experiments.

### 3.2 Ibrutinib treatment impairs HCs *in vitro* and *in vivo*

**Figure 2:**
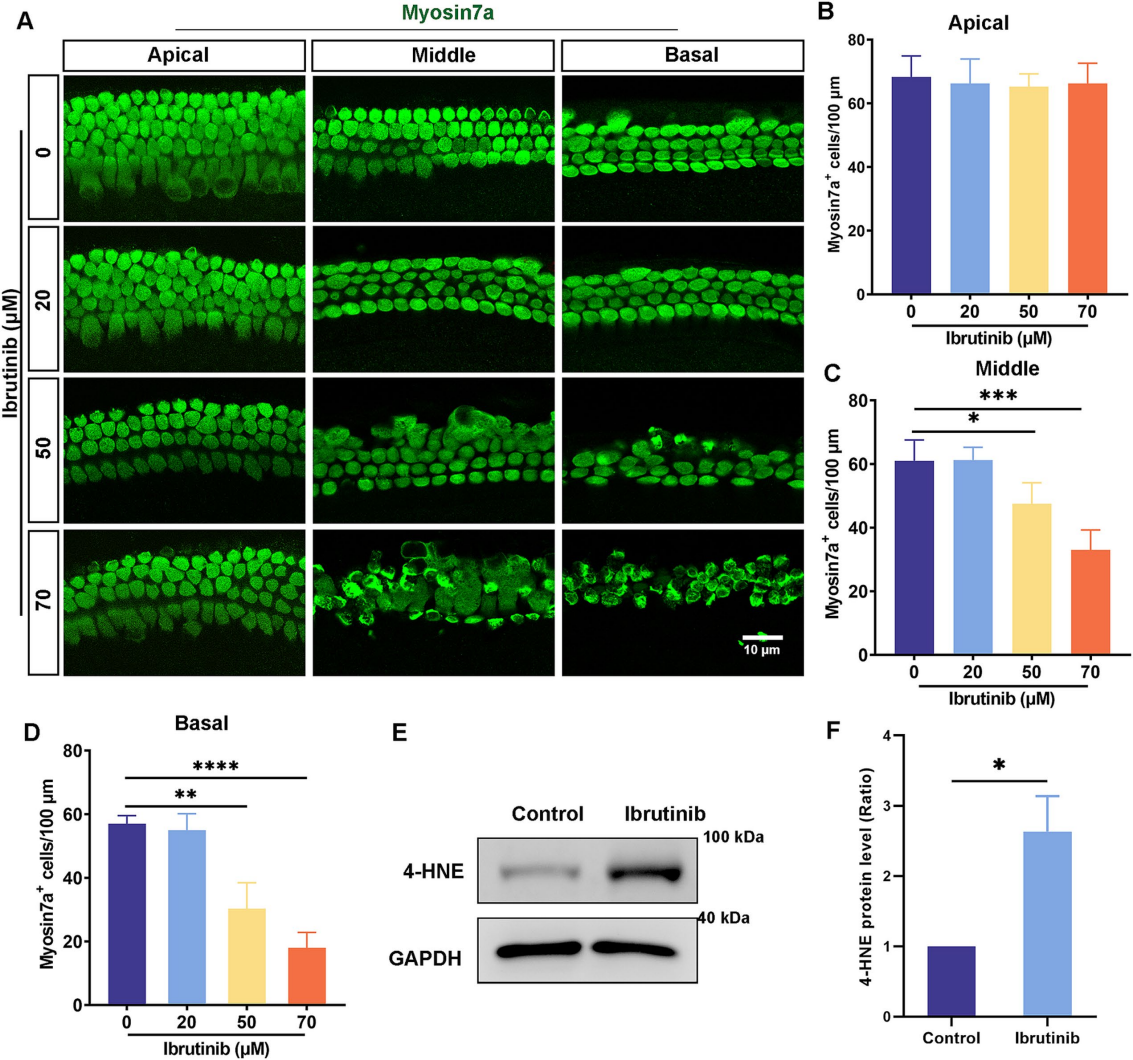
Increased HC loss in explant cultures after treatment with ibrutinib. **(A)** Immunofluorescence analysis with myosin7a showed HC loss in cells treated with 50 and 70 μM ibrutinib for 24 h. Myosin7a-positive HCs (green). Scale bar: 10 μm. **(B–D)** HC quantification along the regions of the cochlea is shown in **(A)**, *n* = 3. **(E)** Examination of the 4-HNE protein level in cochlea through western blotting analysis after treatment with 70 μM ibrutinib for 24 h. **(F)** Statistical assessment of the western blotting data from panel **(E)**, *n* = 3. Data are shown as the mean ± SD. The significance degrees are denoted as follows: * *p* < 0.05, ** *p* < 0.01, *** *p* < 0.001, **** *p* < 0.0001.

Cochlear explants were exposed to 20, 50, and 70 μM ibrutinib for 24 h to explore the ototoxic effects of ibrutinib. Immunofluorescence staining showed no notable damage to the apical turn of HCs after ibrutinib treatment (Figures 2A,B). However, exposure to 50 and 70 μM ibrutinib caused a notable reduction in the quantity of middle and basal turns of HCs (Figures 2A,C,D). These findings suggest that the depletion of cochlear HCs is a consequence of ibrutinib administration. Because 70 μM caused the most serious damage to the HCs, this concentration was selected for subsequent experiments. Additionally, 4-HNE protein levels were measured to assess oxidative stress in HCs after ibrutinib treatment. Western blotting revealed considerably higher 4-HNE protein levels than those in the control group (Figures 2E,F).

**Figure 3:**
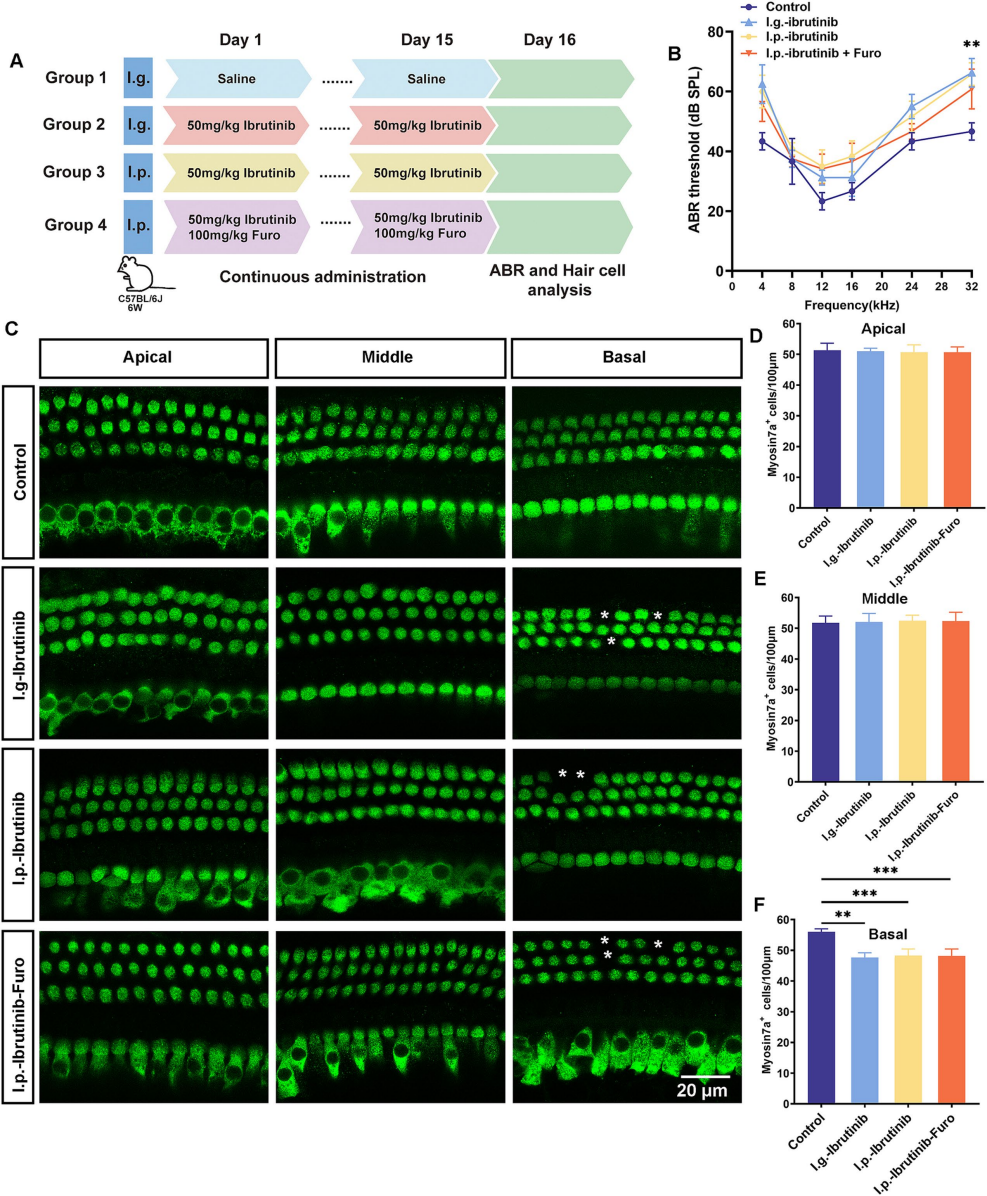
Ibrutinib-induced injury of HCs and hearing loss. **(A)** Schematic diagram illustrating the administration of ibrutinib to mice. **(B)** Evaluation of the mice’s auditory threshold according to the measurements. **(C)** Myosin7a immunofluorescence images of HCs in different regions of the cochlea in the mice treated with oral gavage (I.g.), intraperitoneal (I.p.), and I.p. ibrutinib with furosemide. Each treatment arm had three mice. Scale bar: 20 μm. **(D–F)** Quantification of HCs at 100 μm intervals in the cochlea. Data are shown as the mean ± SD. The significance degrees are denoted as follows: ** *p* < 0.01, *** *p* < 0.001.

To evaluate ibrutinib-induced hearing loss, chronic ibrutinib damage models were constructed using 6-week-old C57BL/6 J mice administered different modalities: oral gavage, intraperitoneal injection alone, or in combination with furosemide (Figure 3A). All ibrutinib administration models experienced high-frequency hearing impairment (Figure 3B). Staining for HCs and counting of myosin7a-positive cells showed considerable deficits in basal cochlear HCs (Figures 3C–F). As no noticeable variations in the ABR results were observed among the three ibrutinib administration models, oral gavage was selected for subsequent experiments.

### 3.3 Ibrutinib disrupts the GPR83–AKT axis in damaged HCs

**Figure 4:**
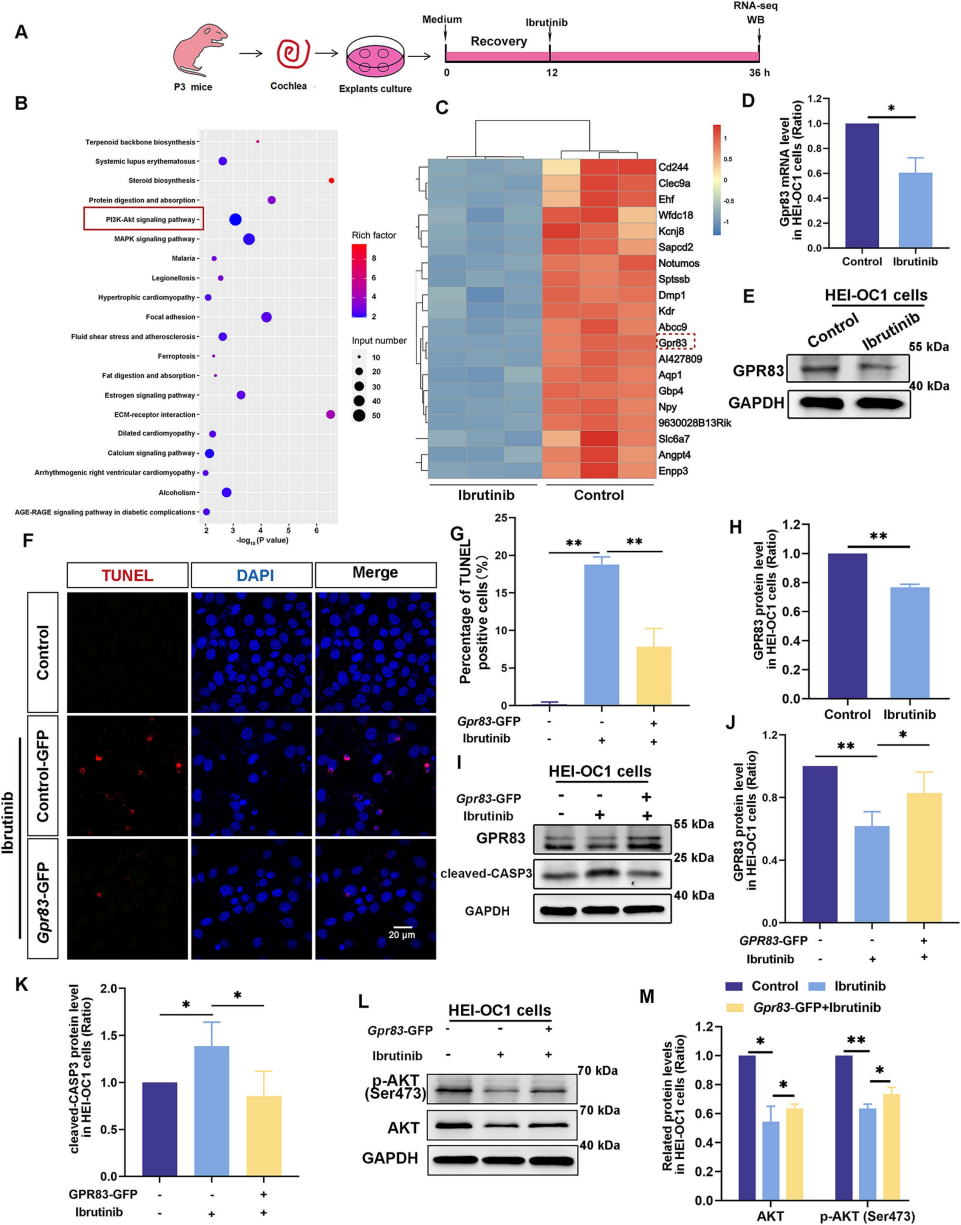
Ibrutinib inhibits GPR83 expression and AKT activation in cochlear HCs and HEI-OC1 cells. **(A)** Flowchart of the RNA sequencing experiment. **(B)** Kyoto Encyclopedia of Genes and Genomes (KEGG) pathway enrichment analysis results. The importance of enrichment is presented on the horizontal axis as the negative logarithm of the *p*-value. Greater values indicate higher enrichment significance. The enriched KEGG pathways are shown on the vertical axis. The size of the dot refers to the quantity of genes participating in splicing within the pathway, whereas the blackness of the dot shows the amount of gene enrichment. The 20 KEGG pathways with the highest ranking based on *p*-value are illustrated. **(C)** Heatmap of the differential gene expression results of the HCs after ibrutinib treatment. **(D)** qPCR results showed *Gpr83* mRNA levels after ibrutinib treatment for 24 h in HEI-OC1 cells, *n* = 3. **(E)** The western blotting results of the GPR83 protein levels after 24 h of ibrutinib treatment in HEI-OC1 cells. **(F)** TUNEL and DAPI staining showing apoptosis of HEI-OC1 cells exposed to ibrutinib for 16 h. HEI-OC1 cells were transfected with *Gpr83*–GFP plasmids for 16 h and treated with 5 μM ibrutinib for 16 h. Scale bar: 20 μm. **(G)** The number of cells positive for DAPI and TUNEL staining from panel **(F)**, *n* = 3. **(H)** Quantitative evaluation of the western blotting results in **(E)**, *n* = 3. **(I)** Western blotting results showing the protein levels of GPR83 and cleaved CASP-3 in HEI-OC1 cells transfected with *Gpr83*-GFP plasmids and treated with ibrutinib for 16 h. **(J,K)** The western blotting bands were quantified in **(I)**, *n* = 3. **(L)** Western blotting results indicate the total AKT and p-AKT protein levels in the HEI-OC1 cells transfected with *Gpr83*–GFP plasmids treated with ibrutinib for 24 h. **(M)** Quantification of the intensity of AKT and p-AKT western blot bands in **(L)**, *n* = 3. Data are shown as the mean ± SD format. The significance degrees are denoted as follows: * *p* < 0.05 and ** *p* < 0.01.

RNA sequencing was used to investigate the potential mechanisms of ibrutinib-induced ototoxicity (Figure 4A), and the PI3K–AKT pathway, which is involved in the deterioration of cochlear HCs, was verified using KEGG analysis (Figure 4B). Ibrutinib altered the expression of the GPCR family member *Gpr83* (Figure 4C). RT-qPCR data analysis revealed a significant reduction in the level of *Gpr83* mRNA in the group treated with ibrutinib compared to the control group (Figure 4D). Western blotting also revealed a notable decrease in both GPR83 protein levels (Figures 4E,H), and the p- AKT expression (Supplementary Figures S1A,B). Western blot analysis also demonstrated a notable decrease in p-EGFR after treatment with ibrutinib in HEI-OC1 cells (Supplementary Figures S1C,D). In addition, immunofluorescence staining confirmed the presence of GPR83 in the cochlear HCs of P3 mice (Supplementary Figure S2).

To investigate the role of *Gpr83* in ibrutinib-damaged HEI-OC1 cells. *Gpr83*–GFP plasmids were used to overexpress GPR83 to determine whether GPR83 reduced ibrutinib-induced injury. TUNEL staining revealed a decrease in the number of apoptotic cells after GPR83 upregulation (Figures 4F,G). Western blot analysis also revealed a substantial decrease in the amounts of cleaved CASP3 protein in HEI-OC1 cells transfected with *Gpr83*–GFP compared to cells treated with ibrutinib alone (Figures 4I–K). These results suggest that GPR83 upregulation protects against ibrutinib-induced cell damage. In HEI-OC1 cells, western blotting revealed elevated AKT protein levels after transfection with *Gpr83*–GFP (Figures 4L,M), suggesting that GPR83 activates the AKT pathway. These findings imply that GPR83 mitigates ibrutinib-induced ototoxicity by enhancing the AKT pathway. Additionally, previous studies suggest that the EGFR-AKT signaling pathway affects cell survival (34, 35). We indeed found the p-EGFR reduced after ibrutinib treatment in HEI-OC1 cells (Supplementary Figure S2).

### 3.4 Z-VAD-FMK protects against ibrutinib-induced apoptosis in HEI-OC1 cells

**Figure 5:**
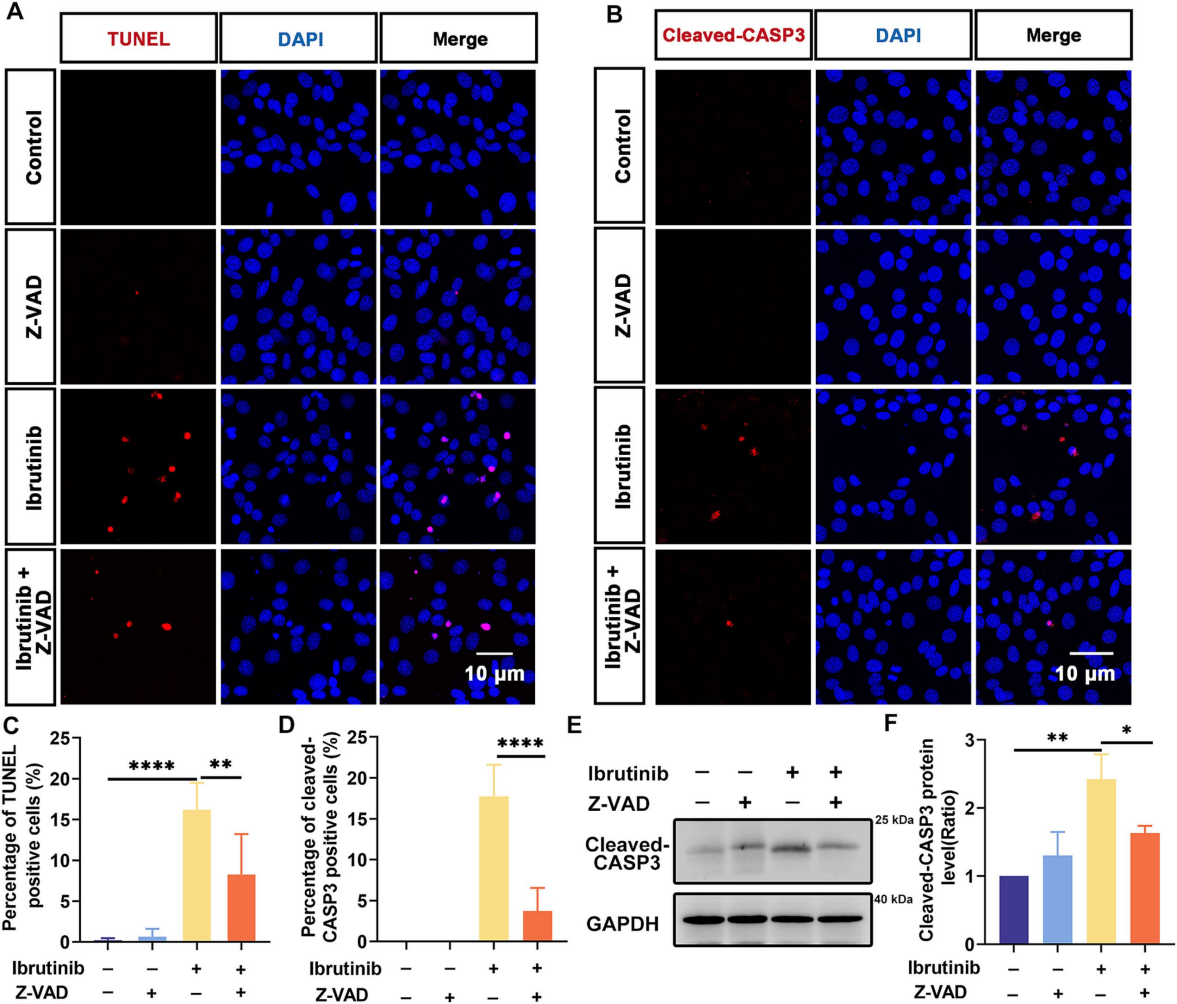
Z-VAD-FMK inhibits HEI-OC1 cell apoptosis after ibrutinib-induced injury. **(A)** TUNEL staining showed positive HEI-OC1 cells after 16 h of Z-VAD-FMK (Z-VAD) and ibrutinib treatment. Scale bar: 10 μm. **(B)** Immunofluorescence staining results using DAPI and cleaved CASP3 antibody. HEI-OC1 cells were treated with Z-VAD-FMK and ibrutinib for 16 h. Scale bar: 10 μm. **(C)** Analysis of the TUNEL and DAPI-positive HEI-OC1 cells in **(A)**, *n* = 3. **(D)** Determination of the cleaved CASP3 positive cells, *n* = 3. **(E)** Examination of the cleaved CASP3 protein expression through western blotting analysis after treatment with ibrutinib and Z-VAD-FMK for 16 h. **(F)** Statistical assessment of the western blotting data from panel **(E)**, *n* = 3. Data are shown as the mean ± SD. The significance degrees are denoted as follows: * *p* < 0.05, ** *p* < 0.01, **** *p* < 0.0001.

The effect of Z-VAD-FMK on ibrutinib-induced HEI-OC1 cell injury was investigated. Cells were exposed to Z-VAD-FMK alone for 24 h at varying concentrations, with no observed toxicity (Supplementary Figure S3A). Subsequently, 50 μM Z-VAD-FMK were selected for further cell protection studies. HEI-OC1 cells were subjected to a 24 h exposure to both ibrutinib and Z-VAD-FMK. The CCK-8 assay demonstrated that treating HEI-OC1 cells with Z-VAD-FMK increased cell viability (Supplementary Figure S3B). Moreover, TUNEL staining revealed a reduction in ibrutinib-induced cell death with the use of Z-VAD-FMK (Figures 5A,C). Cleaved CASP3 staining corroborated the TUNEL assay results (Figures 5B,D). Moreover, western blot analysis demonstrated a significant decrease in cleaved CASP3 expression with Z-VAD-FMK and ibrutinib co-administration compared to ibrutinib treatment alone (Figures 5E,F). These findings support the efficacy of Z-VAD-FMK in protecting HEI-OC1 cells from ibrutinib-induced damage.

### 3.5 Z-VAD-FMK protects HCs against ibrutinib-induced apoptosis

**Figure 6:**
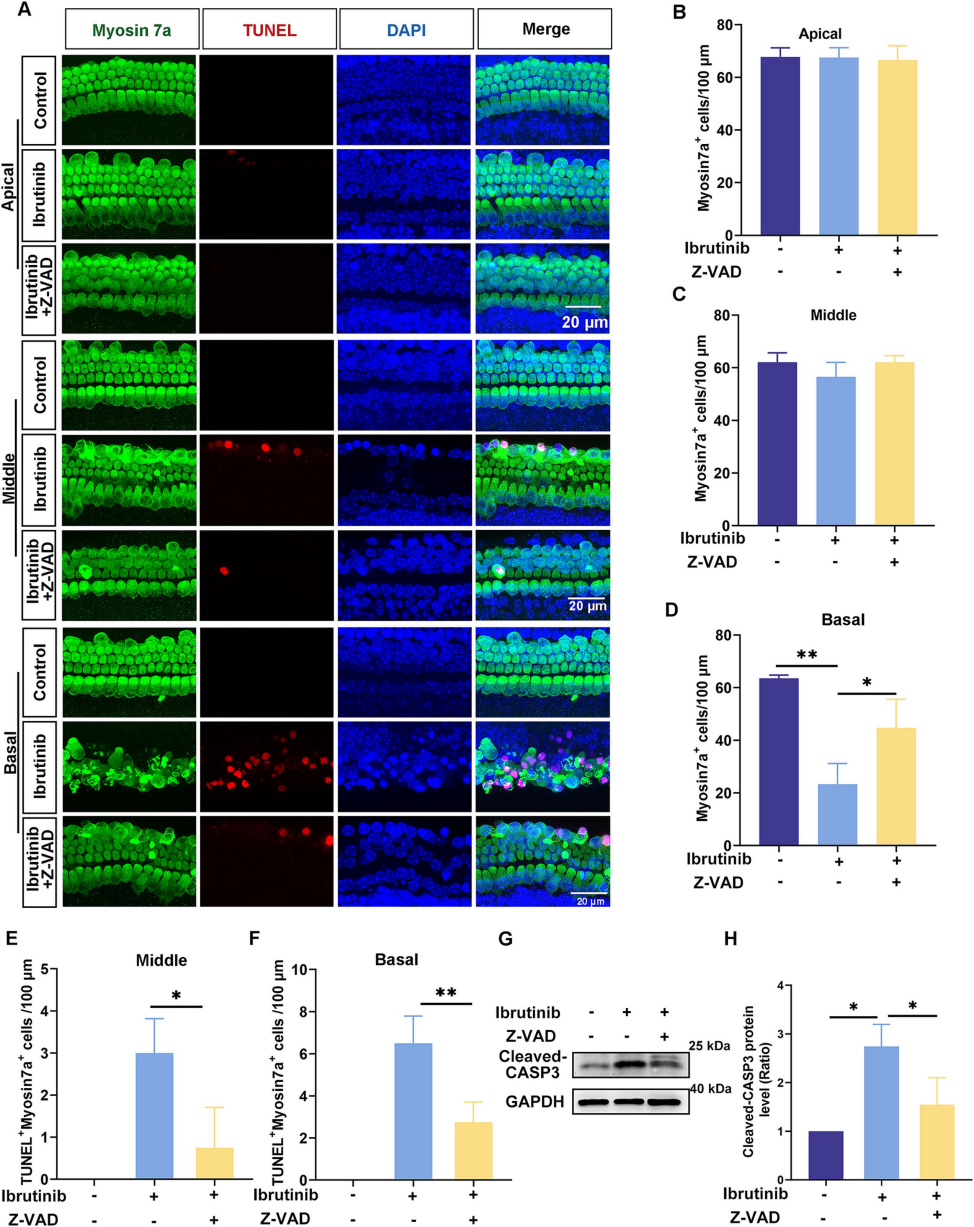
Z-VAD-FMK mitigates ibrutinib-induced HC apoptosis. **(A)** Examination of TUNEL and myosin7a through immunofluorescence in the cochlear apical, middle, and basal region after 16 h of ibrutinib and Z-VAD-FMK administration. Scale bar: 20 μm. **(B–D)** HC quantification among all cochlear regions, *n* = 3. **(E,F)** Measurement of TUNEL-positive HCs in the cochlear apical, middle, and basal region, *n* = 3. **(G)** Assessment of cleaved CASP3 expression changes in cochlear HCs exposed to ibrutinib and Z-VAD-FMK via western blotting analysis. **(H)** Quantitative examination of the western blotting bands of cleaved CASP3 shown in **(G)**, *n* = 3. Data are shown as the mean ± SD. The significance degrees are denoted as follows: * *p* < 0.05, ** *p* < 0.01.

Explant cultures of cochleae extracted from mice were obtained to further examine the protective effects of Z-VAD-FMK against ibrutinib-induced injury in HCs. The cultured explants were first exposed to 0–200 μM Z-VAD-FMK. The immunofluorescence labeling of myosin7a revealed that exposure to 200 μM Z-VAD-FMK for 24 h did not damage HCs (Supplementary Figures S4A,B). Therefore, the explant cultures were exposed to 70 μM ibrutinib and 200 μM of Z-VAD-FMK for 16 h. The quantity of myosin7a-positive HCs was notably increased in the basal regions of the cochlea co-treated with Z-VAD-FMK compared with ibrutinib treated only (Figures 6A–D). Moreover, the quantity of myosin7a and TUNEL double-positive HCs significantly decreased in the middle and basal regions of the cochlea co-treated with Z-VAD-FMK (Figures 6A,E,F). Western blot data obtained from cochlear explants corroborated the immunofluorescence results (Figures 6G,H). These findings provide compelling evidence that Z-VAD-FMK effectively protects HCs from ibrutinib-induced apoptosis.

### 3.6 Z-VAD-FMK attenuates ibrutinib-induced hearing loss

**Figure 7:**
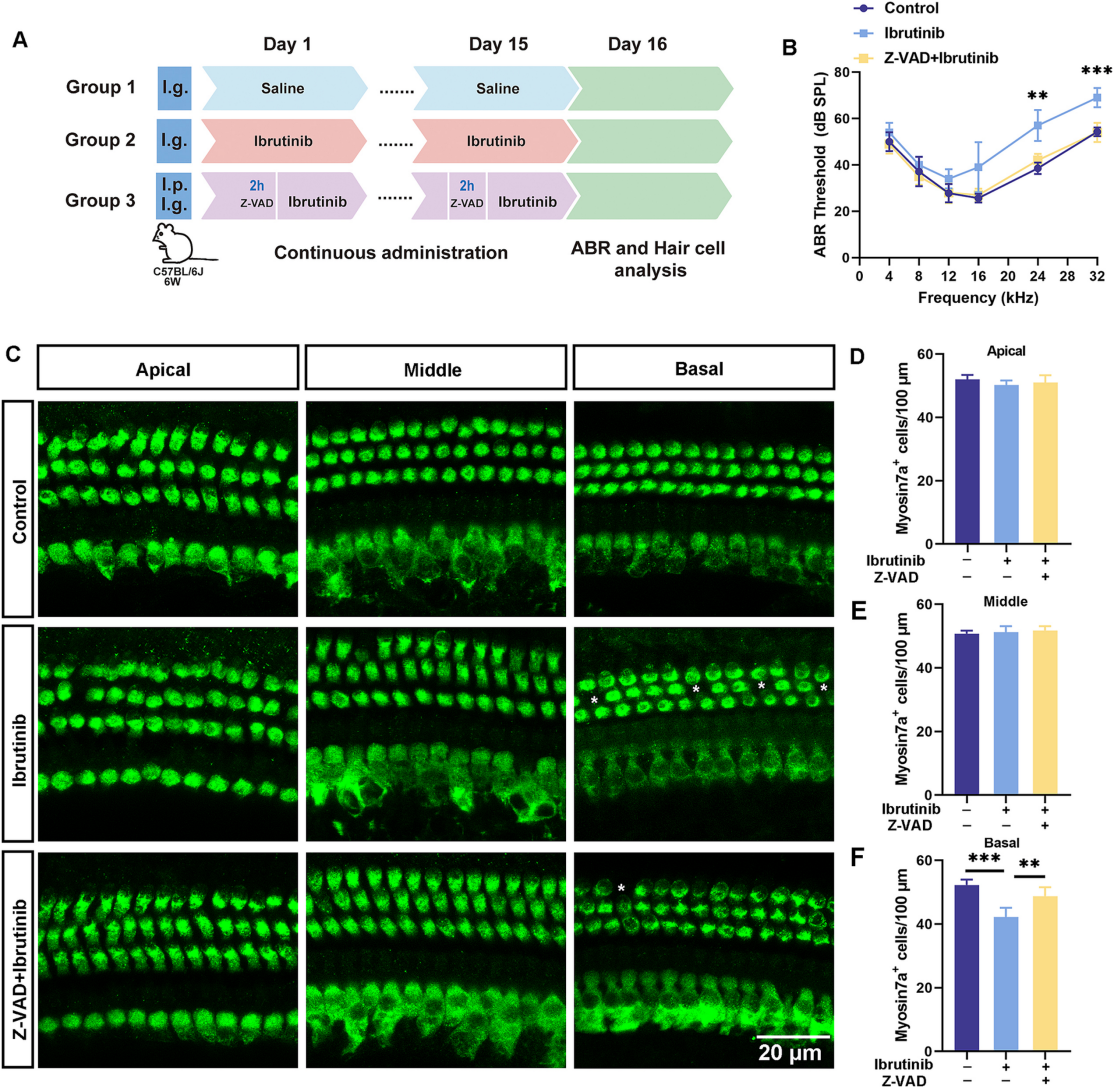
The protective effects of Z-VAD-FMK on cochlear damage and hearing loss induced by ibrutinib. **(A)** Drug administration protocol in mice. **(B)** Results from the ABR tests highlight the effectiveness of Z-VAD-FMK in alleviating ibrutinib-induced auditory dysfunction. **(C)** Visualization of the preserved cochlear HCs using myosin7a immunofluorescence in mice treated with saline (control), ibrutinib (50 mg/kg), and Z-VAD-FMK (2 mg/kg) for 15 days. Scale bar: 20 μm. **(D–F)** Statistical evaluation of myosin7a-positive HCs shows that Z-VAD-FMK can protect against HC depletion in the cochlear regions. Data are shown as the mean ± S.D. format. The significance degrees are denoted as follows: ** *p* < 0.01 and *** *p* < 0.001.

Z-VAD-FMK was administered via intraperitoneal injection to 6-week-old C57BL/6 J mice 2 h before oral gavage with ibrutinib daily for 15 days (Figure 7A). Notably, mice receiving Z-VAD-FMK exhibited enhanced auditory function and greater HC survival than those not receiving Z-VAD-FMK (Figures 7B–F). These findings indicate that Z-VAD-FMK protects against ibrutinib-induced cochlear injury.

**Figure 8:**
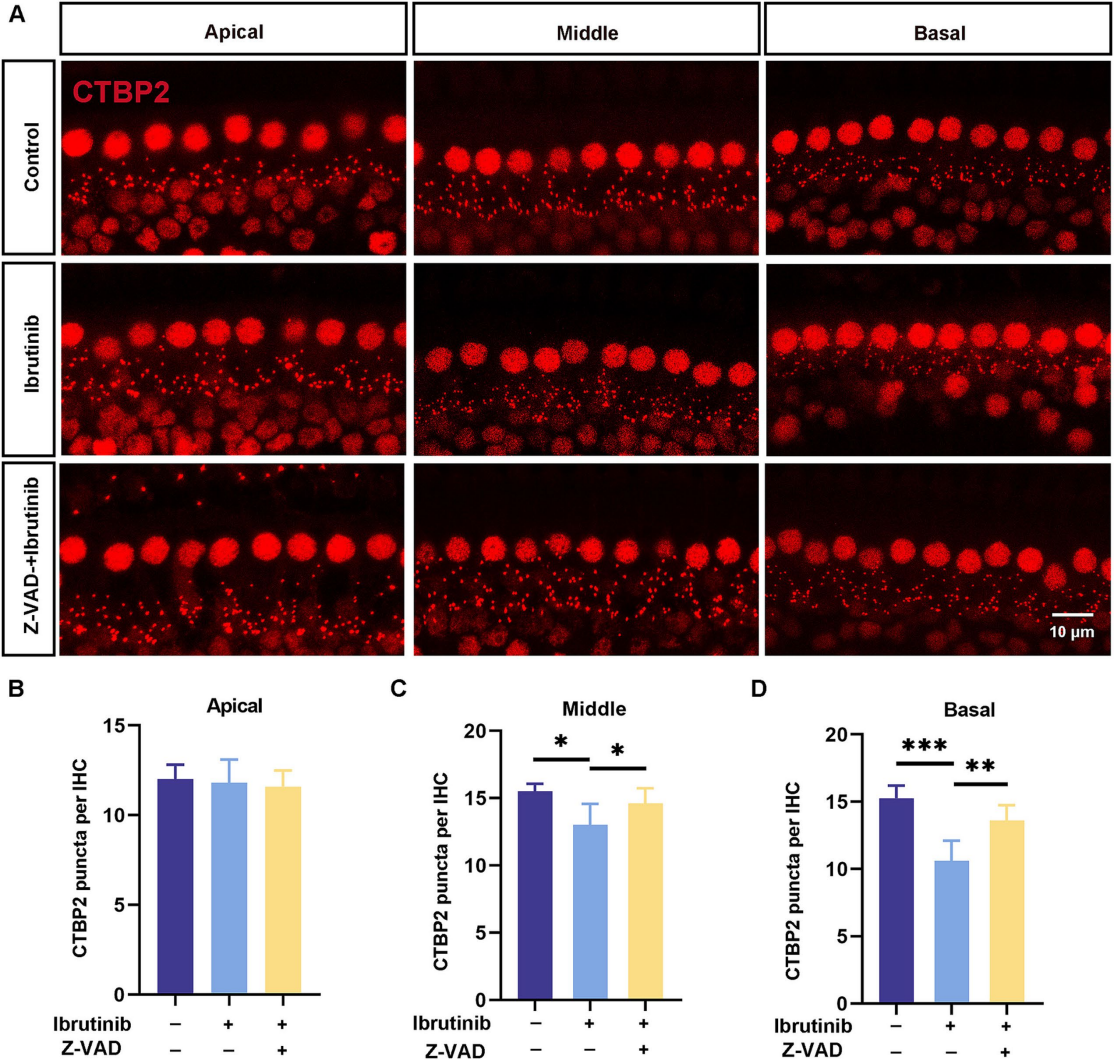
Z-VAD-FMK alleviates ibrutinib-induced ribbon synapse damage. **(A)** Images of CTBP2-labeled pre-synaptic structures in cochlear sections (apical to basal) from C57BL/6 J mice after a 15-day treatment with ibrutinib and Z-VAD-FMK displaying notable distinctions between the control (saline), ibrutinib, and Z-VAD-FMK groups. Ibrutinib dosage: 50 mg/kg; Z-VAD-FMK dosage:2 mg/kg. Scale: 10 μm. **(B–D)** The number of CTBP2 puncta from panel **(A)**, *n* = 3. Each group comprised four mice. Data are shown as the mean ± SD. The significance degrees are denoted as follows: * *p* < 0.05, ** *p* < 0.01 and *** *p* < 0.001.

CTBP2/RIBEYE antibodies were used to identify synapses in ibrutinib-treated HCs to explore the possible effect of ibrutinib on ribbon synapse loss. After ibrutinib exposure, a notable reduction in CTBP2 dots was observed in the middle and basal regions of the cochlea. In contrast, no apparent changes were observed in the apical turn (Figure 8A). However, Z-VAD-FMK administration prevented the synaptic loss, as shown by the retention of pre-synaptic dots (Figures 8B–D). These findings suggest that Z-VAD-FMK alleviates ibrutinib-induced sensorineural hearing loss *in vivo*.

## 4 Discussion

Some cancer-fighting drugs, such as cisplatin, can penetrate the blood–brain barrier, resulting in DNA damage, oxidative stress, and mitochondrial dysfunction within cochlear HCs, leading to programmed cell death (36–38). Ribbon synapses within the ear play an essential role in facilitating the encoding of sound and the release of neurotransmitters by linking the inner HCs with spiral ganglion neurons (39). This study aimed to determine the intricate mechanism by which ibrutinib induces cochlear HC death and confirmed that ibrutinib induces hearing impairment by damaging HCs and synapses within the cochlea (Figures 7, 8), consistent with the outcomes of earlier cisplatin research (40). Besides the well-documented cardiovascular hazards associated with the use of ibrutinib, this drug can bypass the blood–brain barrier and cause neurological damage (41, 42). In the live animal trials, the mode of administration of ibrutinib did not affect ibrutinib-induced hearing loss and HC damage (Figure 3), indicating that it did not affect the capacity of ibrutinib to cross the blood–labyrinth barrier. Consistent with previous findings, the *in vitro* experiments confirmed that ibrutinib could induce cell death, elevate ROS levels, and damage mitochondria in HEI-OC1 cells (Figures 1A–C,G–I). In particular, a notable increase in cleaved CASP3 protein levels was observed, suggesting caspase-3 activation (Figures 1D,E). The phosphorylated Akt protein has a crucial function in protecting HCs by inhibiting cell death (43, 44). In this study, further analysis using RNA-Seq of cochlear HCs treated with ibrutinib demonstrated a decrease in the activation of the PI3K-AKT pathway (Figure 4B). Similarly, Lin et al. demonstrated that ibrutinib prevents the activation of EGFR and hinders the Akt pathway in hepatocellular carcinoma (45).

Mutations in some GPCR family proteins, such as GPR156 and GPR26, can cause hearing loss (46, 47). In this study, differential gene expression data showed that *Gpr83* levels decreased after ibrutinib treatment (Figures 4C–E). Therefore, Class A orphan GPR83 receptors are potentially required for preserving the surviving cochlear HCs. To the best of our knowledge, while similar studies may be ongoing globally, this study is the first to show that ibrutinib can cause hearing impairment by blocking the GPR83 or EGFR–AKT pathways involved in HC death (Figure 4; Supplementary Figure S1). Although the clinical prevalence of ibrutinib-induced ototoxicity is yet to be established, our findings have significant clinical implications, especially for patients undergoing long-term ibrutinib therapy, such as those with chronic lymphocytic leukemia (CLL) or other types of leukemia. We also demonstrated that Z-VAD-FMK successfully inhibited the degeneration of cochlear HCs and synapses (Figures 7, 8), as well as, the superior auditory performance of mice that received Z-VAD-FMK.

Given the significant impact of hearing impairment on a patient’s quality of life, the identification of these molecular pathways offers promising opportunities for preventive and therapeutic strategies. It is essential to recognize the potential risks of hearing loss in patients receiving ibrutinib and consider monitoring for ototoxicity as part of routine clinical care. Future studies should focus on investigating the frequency and severity of ibrutinib-induced ototoxicity in clinical populations, alongside the potential of interventions targeting the GPR83 and EGFR–AKT pathways for mitigating the adverse effects of ibrutinib. Out study lays the groundwork for exploring combined therapeutic approaches that could prevent hearing loss without compromising the efficacy of ibrutinib in treating cancer.

This study has some limitations. First, we treated the mice orally for only 15 days (Figure 3), and it remains unclear whether prolonged treatment would exacerbate the ototoxicity associated with ibrutinib. Additionally, we selected a single concentration of ibrutinib for the *in vivo* experiments, based on prior studies, but the potential variation in ototoxicity at different concentrations has not been explored. Moreover, we did not investigate potential protective strategies to prevent HC damage, particularly through the upregulation of the GPR83 signaling pathway. Previous research has identified EGFR inhibitors as potential targets for noise-induced deafness, offering protection against noise-induced hearing loss by inhibiting the EGFR signaling pathway (48). In our study, we observed a reduction in p-EGFR protein levels following ibrutinib treatment (Supplementary Figures S1C,D). However, whether this reduction contributes directly to ibrutinib-induced ototoxicity remains unclear. Further studies are necessary to determine whether targeting the EGFR-AKT pathway could mitigate ibrutinib-induced HC damage. Additionally, understanding the role of GPR83 in auditory impairment in vivo is crucial for developing potential therapeutic interventions.

In summary, further research is recommended to determine whether modulation of the novel GPR83–AKT pathway could alleviate ibrutinib-induced ototoxicity and whether GPR83 modulation could be used as an alternative therapy to reduce ototoxicity *in vivo*.

## Data Availability

The original contributions presented in the study are publicly available. This data can be found here: https://doi.org/10.6084/m9.figshare.28621208.v1.
