# Supplementary material for: GPR83 protects cochlear hair cells against ibrutinib-induced hearing loss through AKT signaling pathways
Source: Front Med (Lausanne). 2025 Apr 3;12:1579285. doi: 10.3389/fmed.2025.1579285 (PMC12003303; doi:10.3389/fmed.2025.1579285)
Supplement: Supplementary file 1 [file Data_Sheet_1.DOCX]

**Supplementary Materials**

**GPR83 protects cochlear hair cells against ibrutinib-induced hearing loss through AKT signaling pathways**

Yuhua Zhang^1#^, Yun Xiao^1#^, Yongjun Zhu^1#^, Lin Yan^1#^, Nan Cheng^1^, Yongjie Wei^1^, Yanghua Tian^2*^, Wei Cao^1*^, Qiaojun Fang^1*^，Jianming Yang^1*^

1. **Supplementary Figure**


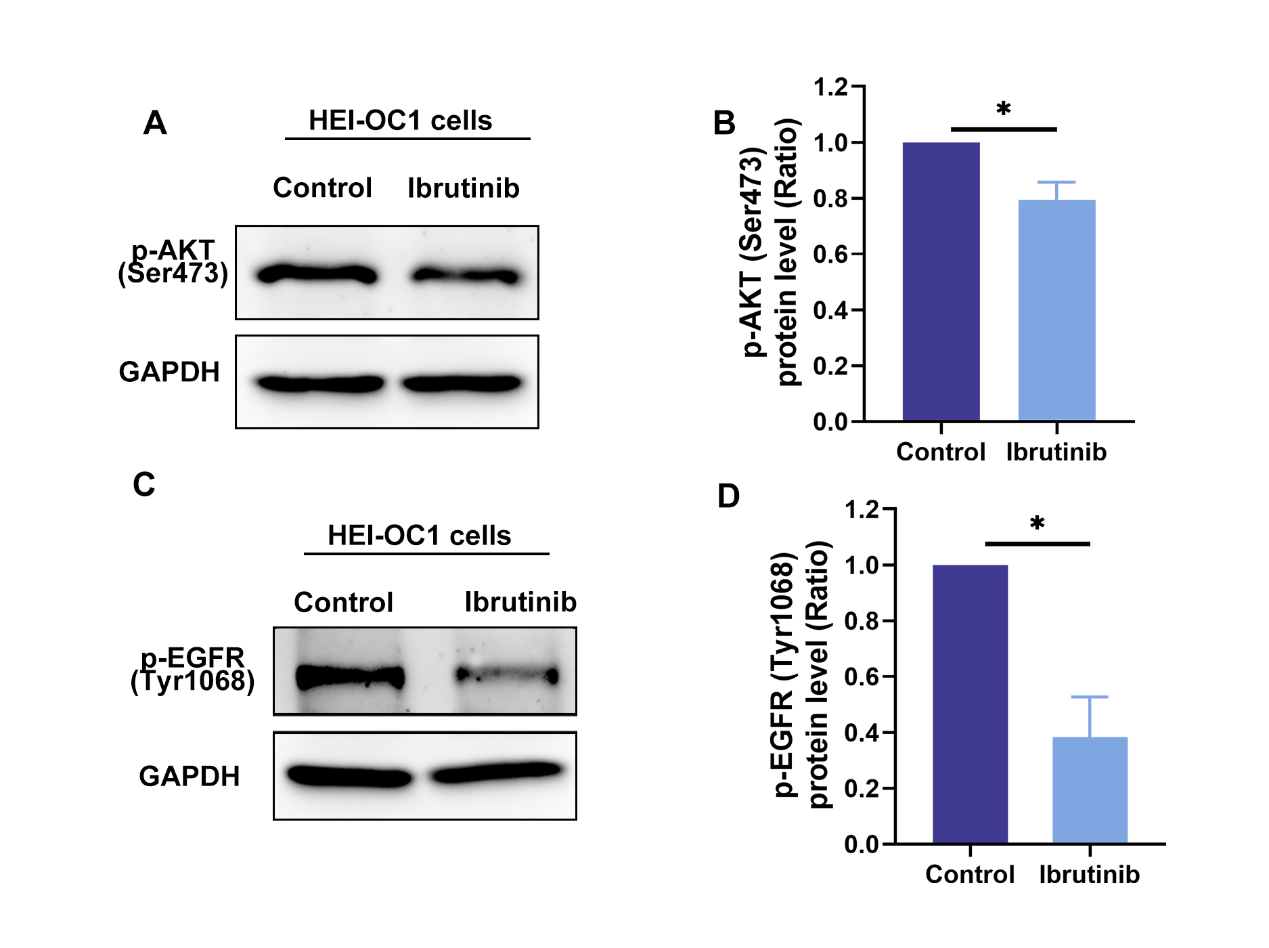


**FIGURE. S1** Changes in p-AKT and p-EGFR protein levels in ibrutinib-treated HEI-OC1 cells. **(A, C)** Western blotting analysis results of p-AKT protein levels in HEI-OC1 cells after 24 h of ibrutinib treatment. **(B**, **D)** Statistical analysis of the western blotting bands of p-EGFR and p-AKT from panels **A** and **C**, n = 3. Data are shown as the mean ± SD format. The significance degrees are denoted as follows: *p < 0.05


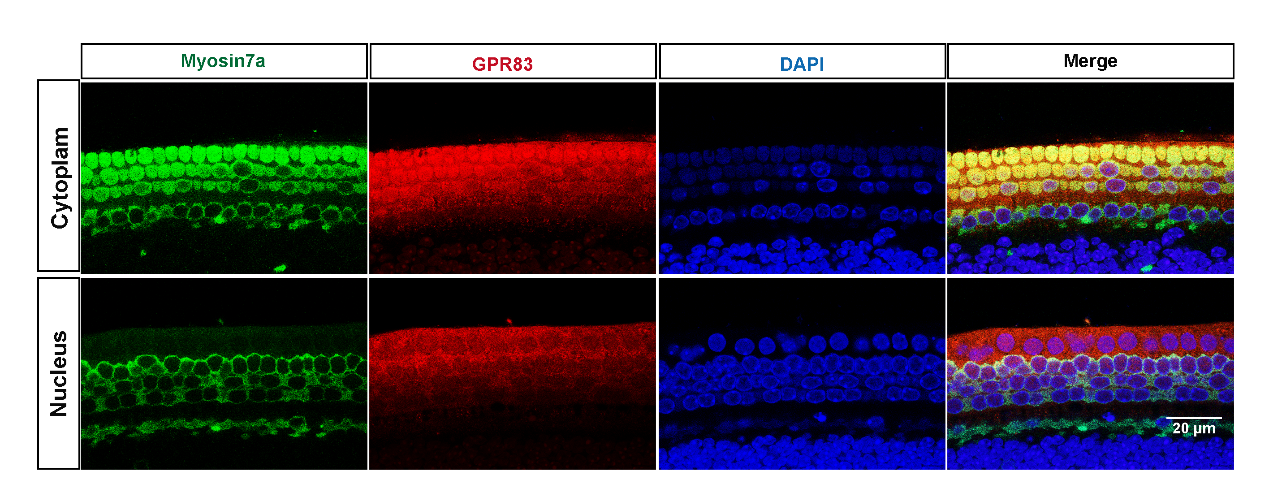


**FIGURE S2.** GPR83 expression pattern in cochlear HCs. Immunofluorescence staining for GPR83 in postnatal day 3 mice cochlear HC.


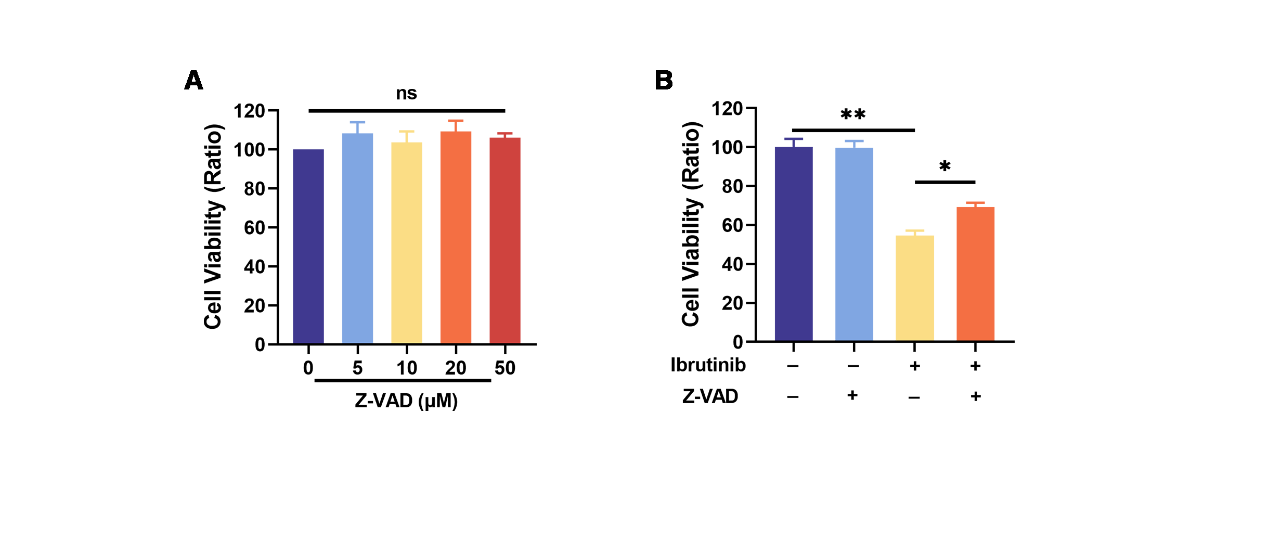


**FIGURE S3** Z-VAD-FMK improves the cell viability of HEI-OC1 cells. **(A)** The cell viability was assessed after 24 h of exposure to various Z-VAD-FMK doses using the CCK-8 kit, n = 3. (**B)** Assessment of cell viability after 24 h of treatment with ibrutinib with or without Z-VAD-FMK, n = 3. Data are shown as the mean ± SD. The significance degrees are denoted as follows: *p < 0.05, **p < 0.01. ns: no significant difference.

**
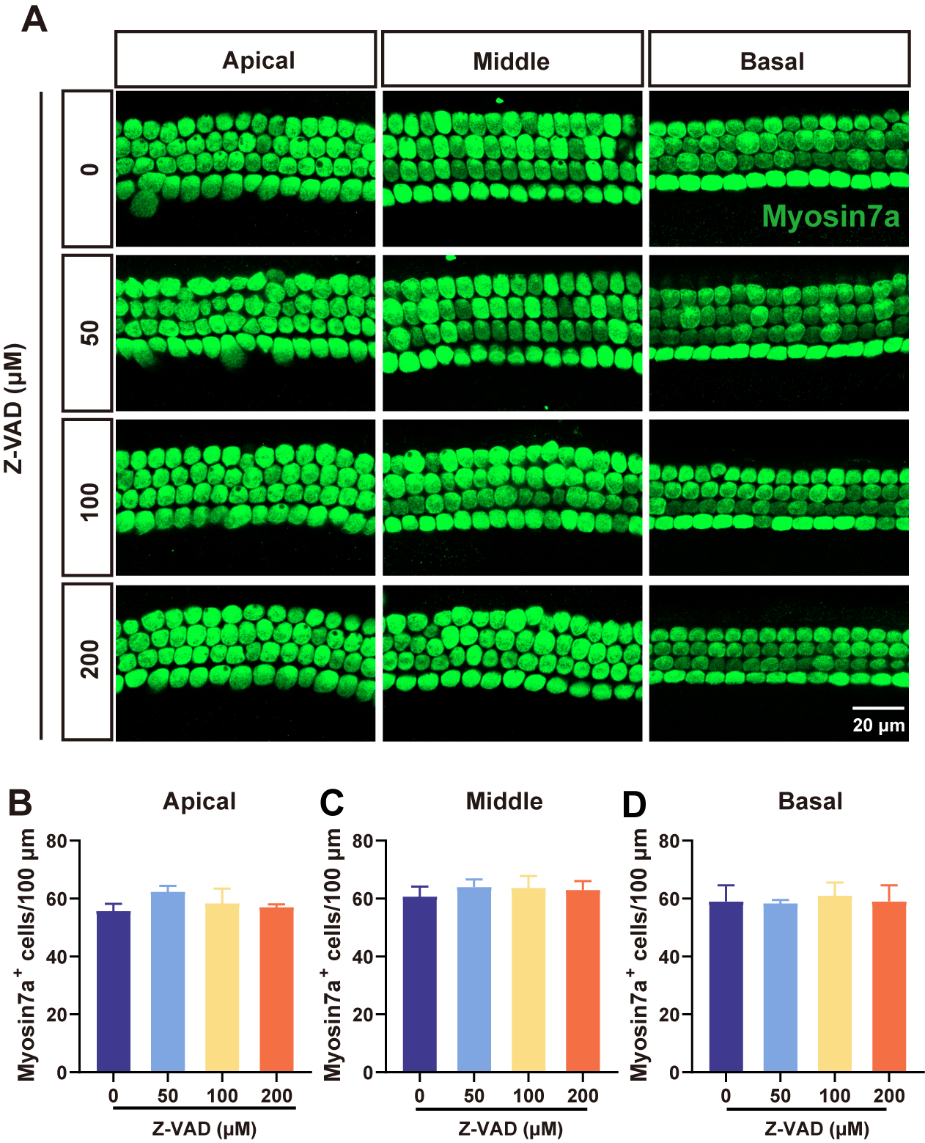
**

**FIGURE S4** Z-VAD-FMK concentration screening. (**A)** Immunofluorescence analysis with myosin 7a showed no HC loss after treatment with varying Z-VAD-FMK concentrations for 24 h. Scale bar: 20 μm. (**B**–**D)** HC quantification among all cochlear regions, n = 3.

1. **Supplementary Table**

**Table S1 Primer sequences**

| **Gene Name** | **Direction** | **Sequences** |
| --- | --- | --- |
| ***Apaf1*** | Forward | AGTAATGGGTCCTAAGCATGTTG |
|  | Reverse | GCGATTGGGAAAATCACGTAAAA |
| ***Caspase-9*** | Forward | TCCTGGTACATCGAGACCTTG |
|  | Reverse | AAGTCCCTTTCGCAGAAACAG |
| ***Caspase-3*** | Forward | ATGGAGAACAACAAAACCTCAGT |
|  | Reverse | TTGCTCCCATGTATGGTCTTTAC |
| ***Gpr83*** | Forward | TCTTCTGTCCTCAGTGCGAG |
|  | Reverse | CAGTCAGAGAAAGTGTAGTTGGC |
| ***Gapdh*** | Forward | AGGTCGGTGTGAACGGATTTG |
|  | Reverse | TGTAGACCATGTAGTTGAGGTCA |
