# Supplementary figures and images for: GPR83 protects cochlear hair cells against ibrutinib-induced hearing loss through AKT signaling pathways
Source: Front Med (Lausanne). 2025 Apr 3;12:1579285. doi: 10.3389/fmed.2025.1579285 (PMC12003303; doi:10.3389/fmed.2025.1579285)

Fig.1D

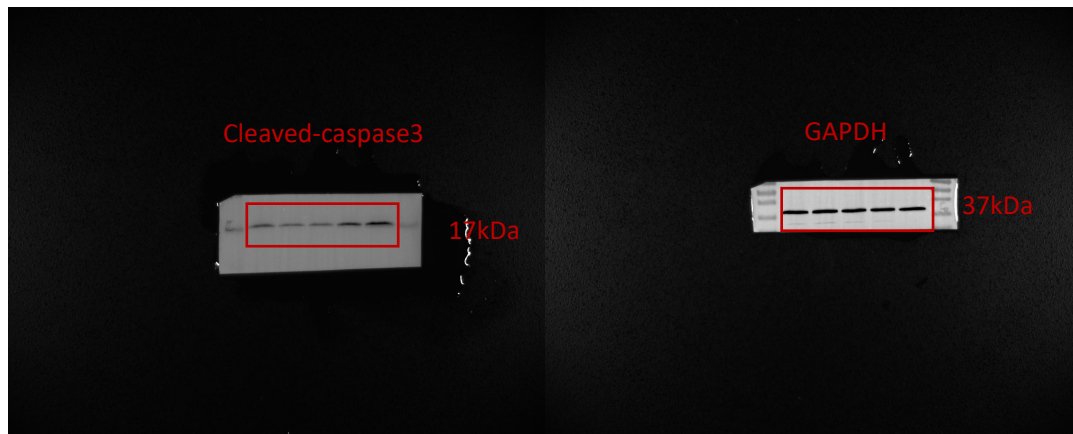

Fig.2E

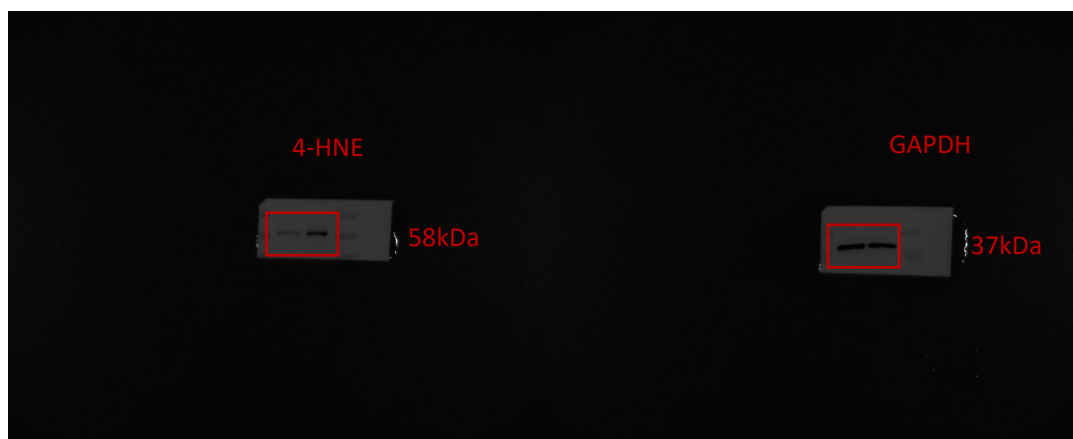

Fig.4E

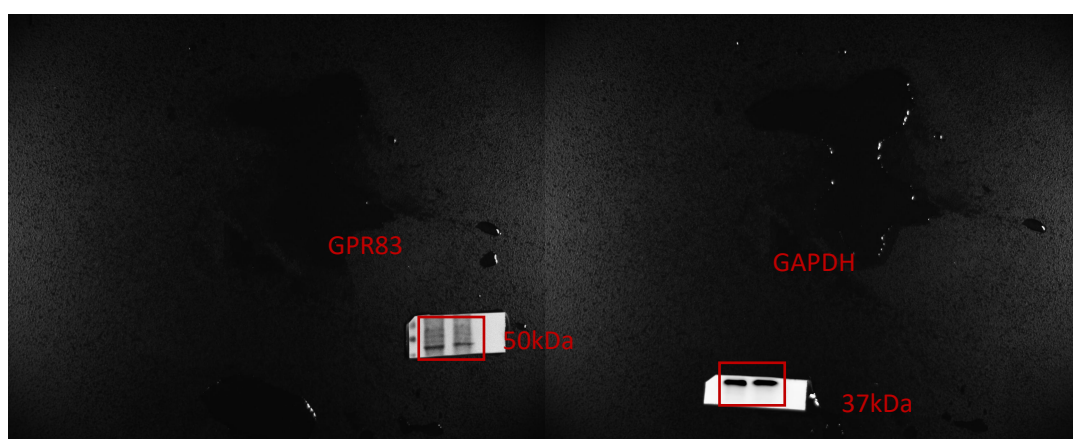

Fig.4I

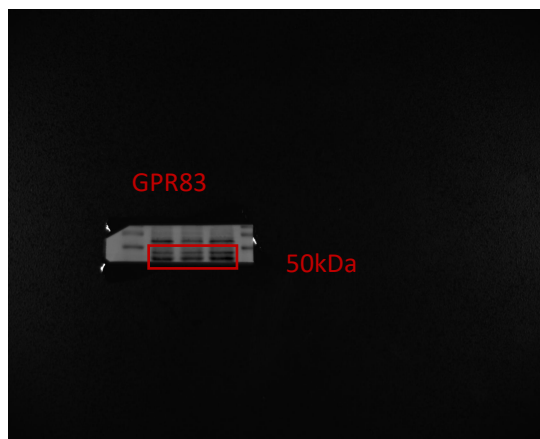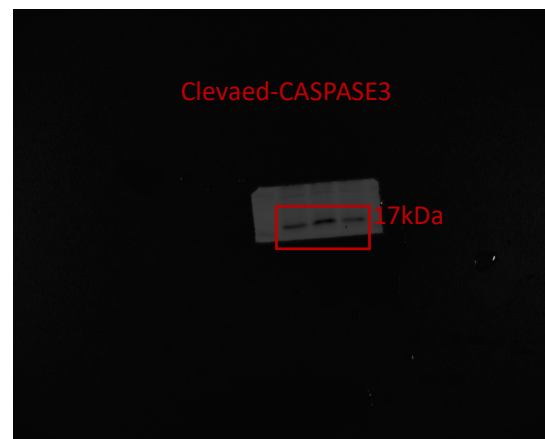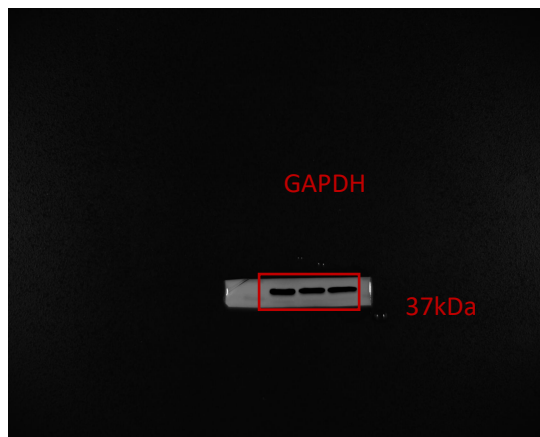

Fig.4L

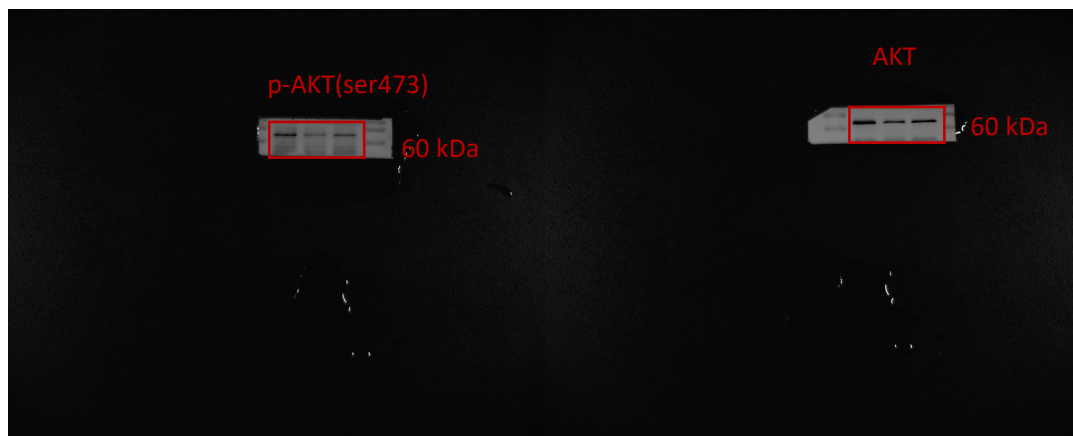

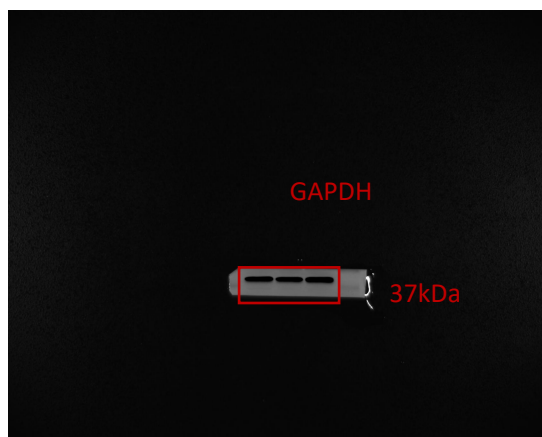

Fig.5E

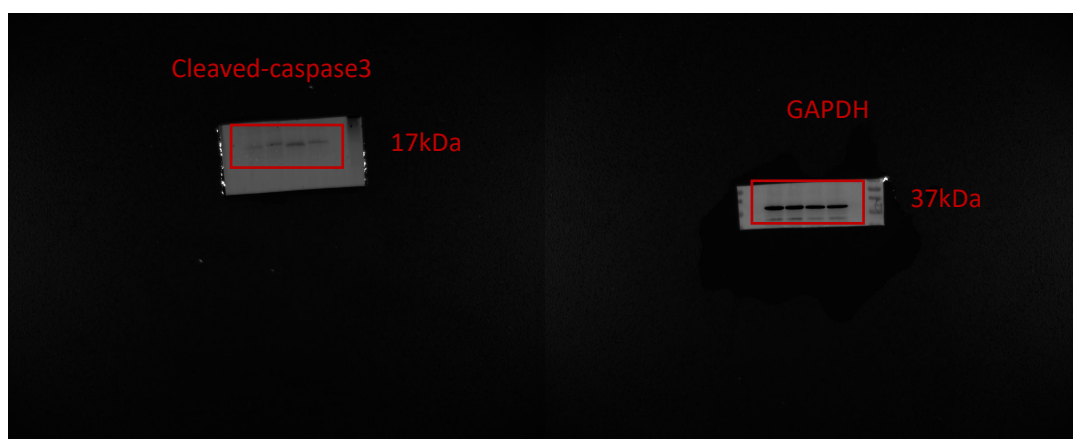

Fig.6G

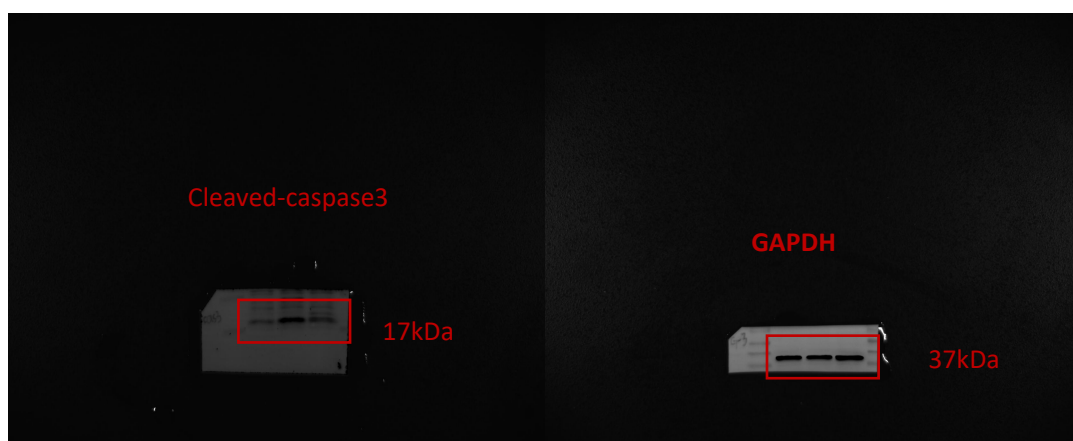

Fig.S1AB

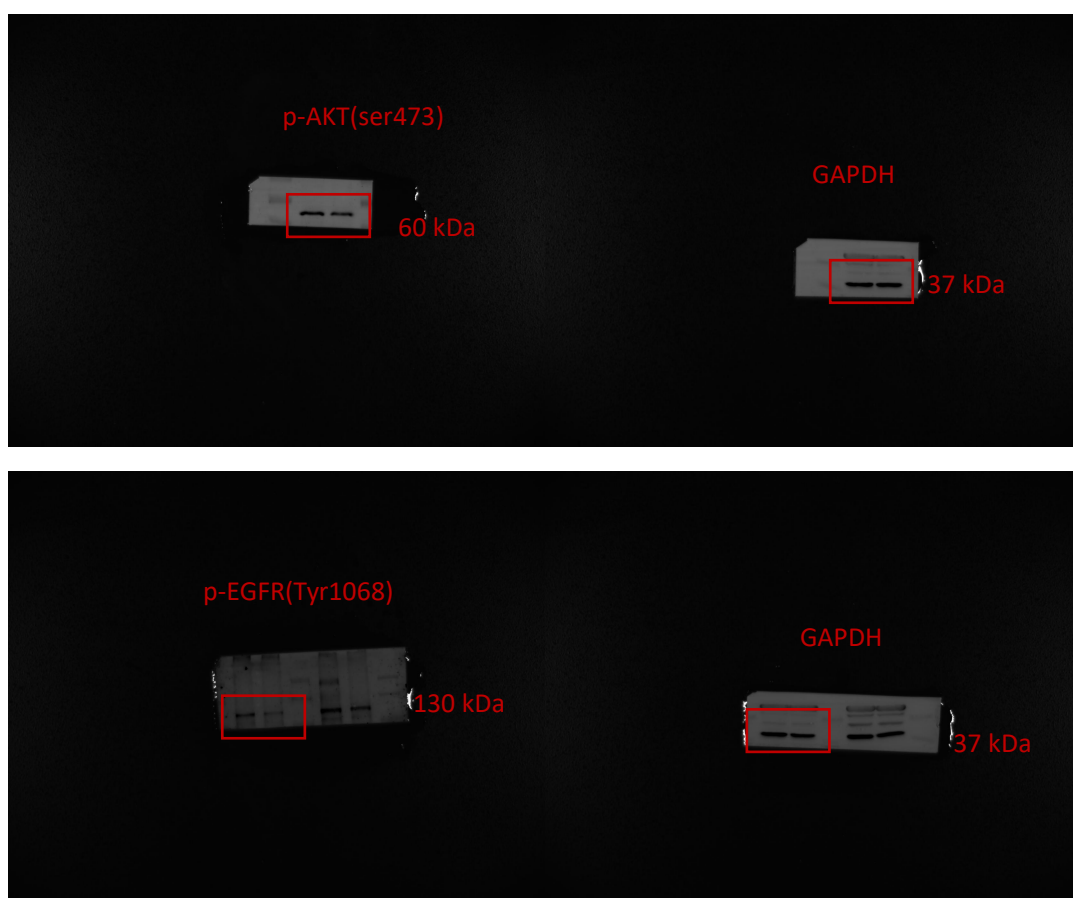

Supplement: Supplementary file 2 [file Image_1.PDF]
